# Clinical manifestations and approach to the management of patients with common variable immunodeficiency and liver disease

**DOI:** 10.3389/fimmu.2023.1197361

**Published:** 2023-06-05

**Authors:** Vanessa Daza-Cajigal, Marina Segura-Guerrero, María López-Cueto, Ángel Robles-Marhuenda, Carmen Camara, Teresa Gerra-Galán, Ricardo Gómez-de-la-Torre, Carmen L. Avendaño-Monje, Silvia Sánchez-Ramón, María J. Bosque-Lopez, Adriana Quintero-Duarte, María L. Bonet-Vidal, Jaime Pons

**Affiliations:** ^1^ Department of Immunology, Hospital Universitario Son Espases, Palma, Spain; ^2^ Research Unit, Balearic Islands Health Research Institute (IdISBa), Palma, Spain; ^3^ Department of Internal Medicine, Hospital Universitario La Paz, Madrid, Spain; ^4^ Department of Immunology, Hospital Universitario La Paz, Madrid, Spain; ^5^ Department of Clinical Immunology, Instituto de Medicina del Laboratorio (IML), Hospital Clínico San Carlos, Madrid, Spain; ^6^ Autoimmune and Systemic Diseases Unit, Hospital Universitario Central de Asturias, Oviedo, Spain; ^7^ Department of Immunology, Hospital Universitario Central de Asturias, Oviedo, Spain; ^8^ Department of Gastroenterology, Hospital Universitario Son Espases, Palma, Spain; ^9^ Department of Pathology, Hospital Universitario Son Espases, Palma, Spain

**Keywords:** common variable immunodeficiency (CVID), liver disease, nodular regenerative hyperplasia (NRH), immune dysregulation, portal hypertension (PHTN), lymphoproliferation

## Abstract

**Purpose:**

The clinical spectrum of common variable immunodeficiency (CVID) includes predisposition to infections, autoimmune/inflammatory complications and malignancy. Liver disease is developed by a proportion of patients with CVID, but limited evidence is available about its prevalence, pathogenesis and prognostic outcome. This lack of evidence leads to the absence of guidelines in clinical practice. In this study, we aimed at defining the characteristics, course and management of this CVID complication in Spain.

**Methods:**

Spanish reference centers were invited to complete a cross-sectional survey. Thirty-eight patients with CVID-related liver disease from different hospitals were evaluated by a retrospective clinical course review.

**Results:**

In this cohort, abnormal liver function and thrombocytopenia were found in most of the patients (95% and 79% respectively), in keeping with the higher incidence of abnormal liver imaging and splenomegaly. The most common histological findings included nodular regenerative hyperplasia (NRH) and lymphocytic infiltration, which have been associated with portal hypertension (PHTN) leading to a poorer prognosis. Autoimmune/inflammatory complications occurred in 82% of the CVID patients that developed liver disease and 52% of the patients treated with immunomodulators showed a reduction in the liver function tests’ abnormalities during treatment. Among the experts that conducted the survey, there was 80% or more consensus that the workup of CVID-related liver disease requires liver profile, abdominal ultrasound and transient elastography. The majority agreed that liver biopsy should be essential for diagnosis. There was 94% consensus that endoscopic studies should be performed in the presence of PHTN. However, there was 89% consensus that there is insufficient evidence on the management of these patients.

**Conclusion:**

Liver disease varies in severity and may contribute substantially to morbidity and mortality in patients with CVID. Hence the importance of close follow-up and screening of this CVID complication to prompt early targeted intervention. Further research is needed to evaluate the pathophysiology of liver disease in patients with CVID to identify personalized treatment options. This study emphasizes the urgent need to develop international guidelines for the diagnosis and management of this CVID complication.

## Introduction

Common variable immunodeficiency (CVID) is the most prevalent symptomatic primary immunodeficiency (PID) in adult age and is characterized by hypogammaglobulinemia (low levels of IgG and either IgA or IgM isotypes) and impaired antibody response (1–3). The clinical spectrum of CVID includes predisposition to infections, manifestations of immune dysregulation, such as autoimmunity, granulomatosis, lymphoid hyperplasia, enteropathy, and malignancies (3, 4). CVID includes multiple disorders leading to the failure of B-cell responses. Abnormalities in immune cells have been described, including the reduction of class-switched memory B cells, the expansion of transitional B cells and/or CD21low B cells, the reduction of naive T cells and regulatory T cells (Treg), among others (5, 6). The development of next-generation sequencing (NGS) has also shown different genes associated with a CVID-phenotype (7, 8).

The standard CVID treatment is human IgG replacement reducing the frequency of infections and improving the prognostic outcome of these patients (9). However, immunoglobulin replacement has no proven effectiveness on immune dysregulation related complications that have become the major cause of morbidity and mortality in CVID patients (10, 11). Immune dysregulation-related complications such as autoimmune cytopenia, polyclonal lymphoproliferation and granulomatous disease, also involve the gastrointestinal tract leading to complications as malabsorption, gut microbial translocation and liver disease (12, 13).

CVID-related liver disease may be associated to immune dysregulation, but also infection and malignancy. Liver involvement could be defined as a disruption of liver function or portal hemodynamic and may be identified through biochemical, clinical, imaging and histologic diagnostic tools. However, limited evidence is available on the prevalence, pathogenesis and prognosis of liver disease in CVID and there is no standardized monitoring strategy for these patients (3, 13–19). Around 50% of CVID patients have been reported to display a persistent increase of liver enzymes associated with mild hepatomegaly. However, the nature of liver involvement has not been systematically investigated in the majority of CVID cohort studies published (3, 17, 19). CVID patients with liver diseases had been shown to have reduced survival (20), but prevalence varies depending on the detection strategy (3, 21). This lack of evidence leads to the absence of guidelines related to diagnosis and management of CVID-associated liver disease in clinical practice.

Nodular regenerative hyperplasia (NRH) is generally considered the most typical form of liver involvement in CVID. NRH is a histopathologic picture that is thought to be the result of an intra-hepatic vasculopathy, leading to both hepatocyte injury and regeneration. NRH may involve a significant alteration of the blood flow through portal system and has the capacity to progress resulting in portal hypertension (PHTN). Patients with PHTN may develop splenomegaly, ascites, varices, liver dysfunction and hepatopulmonary shunting (1, 3). Laboratory signs of NRH may not be detectable at the beginning of the disease. However, most patients show increased alkaline phosphatase (ALP) and gamma-glutamyl-transpeptidase (GGT) levels, and the clinical signs are the result of non-cirrhotic PHTN due to sinusoidal compression (18). CVID patients with NRH are also more likely to present immune-dysregulation related complications (19), suggesting a possible role for immunomodulatory therapy in halting the progression of liver damage in these patients.

In this study, we report the epidemiology, prevalence of other comorbidities, the use of immunomodulatory treatments, and outcome of our cohort of patients with CVID-related liver disease. We summarize the evidence on the features of liver involvement and diagnostic tools employed in the detection and monitoring among these patients, to better understand and manage this CVID complication.

## Methods

Patient population: Patient data from four different Spanish hospitals were evaluated by a retrospective clinical course review. Experts from each centre included anonymous information of their cohort of patients with CVID related liver disease, such as time and mode of presentation, organ or system involvement, blood parameters and imaging, analysis of liver histology, management and outcomes. We evaluated 38 CVID patients with liver disease and we also included in the study a control group of 55 CVID patients without liver disease that had normal liver biochemistry and imaging.

We applied the diagnostic criteria of the European Society for Immunodeficiency (ESID) for CVID: low levels of IgG and either IgM or IgA isotypes (at least 2 standard deviations below the mean for age or below 5 g/L for adults in at least 2 measurements more than 3 weeks apart); onset of immunodeficiency at an age of 4 or more than 4 years old; absence of isohemagglutinins and/or poor response to vaccines and/or absence or low switched memory B-cells; and other causes of hypoimmunoglobulinemia should be excluded) (1). Evidence of liver disease was considered in CVID patients with clinical, laboratory (abnormal liver function tests (LFTs) for a minimum of 6 months in at least 2 measurements), radiological or histological signs of liver damage or PHTN. Alcoholic hepatitis was excluded.

A structured survey containing statements on diagnosis and management was developed for this study by a committee composed of two immunologists and two hepatologists. Spanish centers that have an Immunology department with experience in treating patients with PID were contacted through the Spanish Society for Immunology (SEI). The participants completed the questionnaire via a web link. This panel indicated their level of agreement on a scale of 1–5, with 1 reflecting “strongly disagree” and 5 “strongly agree”. Responses were graded, unless stated otherwise, as strongly disagree, tend to disagree, neither agree nor disagree, tend to agree and strongly agree. The percentages of the different categories 1 and 2 (disagreement) and 4 and 5 (agreement) were calculated. Unanimity was defined as 100% of the experts agreeing with the recommendation/conclusion (strongly or tend to). Agreement or disagreement was defined as 80% or greater consensus to agree (strongly or tend to) or disagree (strongly or tend to). Majority, when more than 65% but less than 80% of the experts agree or disagree.

Statistical analysis: Data were analysed in GraphPad Prism. Quantitative data were described in terms of median values. Qualitative data were reported in terms of absolute frequencies and percentage. Associations were determined using the Fisher’s exact test and Chi^2^ test. Two-sided *p <*0.05 were considered significant.

Ethical considerations: The authors declare that the research was conducted in the absence of any commercial or financial relationships that could be construed as a potential conflict of interest. Patient data will be used in accordance with the Declaration of Helsinki and ethical approval from the Research Ethics Committee of the Balearic Islands (CEI-IB).

## Results

### 1-Characteristics of CVID patients with liver disease

We evaluated the management and outcomes of a cohort of patients with CVID and liver disease from four Spanish centers. We analyzed age, sex, time and mode of presentation, involvement of other organs, liver blood parameters, imaging studies, cellular immunity by flow cytometry data, liver histology, treatment, morbidity and mortality.

Thirty-eight CVID patients with liver disease were identified, of whom 47% were female and 53% were male patients. The mean age was 50.3 years (SD 13.7 years). The mean age at diagnosis of CVID was 36.3 years (SD 16.5 years) and the mean age of onset of liver disease was 43.4 years (SD 13.3 years), suggesting that liver disease is often a late complication of CVID. The mean age of the group of patients without liver disease was 48.8 years (SD 17.5 years) and the mean age at diagnosis of CVID was 38.9 years (SD 17.1 years).

The cohort of CVID patients with liver disease most frequently presented with recurrent respiratory tract and gastrointestinal (GI) infections at the time of diagnosis of CVID (86%). Other non-infectious complications such as autoimmune cytopenia, lymphoproliferative disease and CVID enteropathy were present in 47% of patients as initial manifestation of the disease.

Liver disease in CVID may be due to infectious, autoimmune, or neoplastic causes. Given that serological tests based on determination of antibodies are unreliable in these patients, HBV and/or HCV infection should be confirmed by the presence of HBsAg and HBV-DNA or HCV-DNA. The presence of autoimmune hepatitis should be confirmed by histological findings. In our cohort of patients, liver involvement was mainly non-infectious. Only three patients had HBV and autoimmune hepatitis was reported in one patient.

### Signs that should prompt a suspicion of liver disease in patients with CVID

CVID patients with liver disease most frequently presented with abnormal LFTs (76%) and abnormal abdominal ultrasound (68%) at the onset of liver disease. LFTs, splenomegaly and abnormal liver imaging were mainly observed as initial signs of suspected liver disease.

### Liver function test pattern and full blood count

Abnormal LFTs were found in 95% of patients with liver disease and in 50% of them this abnormality was progressive (**Table 1**).

**Table 1:** Characteristics of CVID patients with or without liver disease.

Characteristics	CVID patients without liver disease n = 55 (%)	CVID patients with liver disease n = 38 (%)	P value
Mean age (y)	48.8 (SD 17.5)	50.3 (SD 13,7)	
Recurrent respiratory tract infections	47 (85%)	30 (79%)	ns
Recurrent GI infections	16 (29%)	15 (39%)	ns
GLILD	2 (4%)	7 (18%)	ns
Autoimmune cytopenia	8 (15%)	7 (18%)	ns
Thrombocytopenia	5 (9%)	30 (79%)	<0.0001
Splenomegaly	10 (18%)	33 (89%)	<0.0001
CVID enteropathy	16 (29%)	17 (45%)	ns
Lymphoproliferation	14 (25%)	28 (74%)	<0.0005
Non-clonal	8 (15%)	20 (52%)	<0.0001
Clonal	6 (11%)	8 (21%)	ns
Autoimmune/inflammatory complications	30 (55%)	31 (82%)	0.014
Abnormal liver biochemical test at the onset of liver disease	0 (0%)	29 (76%)	
Liver biochemical test	D= 55 (100%)	A= 19 (50%)	
		B= 7 (18%)	
		C= 12 (32%)	
		D= 0 (0%)	
		Total abnormal: 36 (95%)	
Imaging US liver	Normal liver= 55 (100%)	Heterogeneous= 19 (50%)	
		Normal liver = 13 (34%)	
		Hepatomegaly= 8 (21%)	
		Nodules= 6 (16%)	
		Total abnormal: 25 (66%)	
Transient elastography > 6.5 kPa	Not performed = 55 (100%)	30 (81%)	
Histology	Not performed = 55 (100%)	Performed= 25 (66%)	
		NRH = 12 (48%)	
		Lymphocytic infiltration= 17 (67%)	
		Lobular/interface hepatitis= 6 (24%)	
		Periportal/sinusoidal fibrosis=5 (20%)	
		Granuloma = 9 (36%)	
Liver disease complications			
Portal hypertension		23 (61%)	
Ascites		10 (26%)	
Gastric/oesophageal varices		15 (39%)	
GI bleeding		3 (8%)	
Cirrhosis		0 (0%)	
Crude mortality ratio	2 (4%)	8 (21%)	0.013
Mortality associated with liver disease		5 of 8 (63%)	

CVID, common variable immunodeficiency; GLILD, granulomatous-lymphocytic interstitial lung disease; NRH, nodular regenerative hyperplasia; LFTs, liver function test; GI, gastrointestinal; US, ultrasound; NS, not significant; Liver biochemical test pattern, A, progressive elevation; B, persistently abnormal; C, variable; D, normal.

Thrombocytopenia was present in 79% of patients with liver disease compared with 9% of those without (OR 41.25;95%CI 12 to 125.9; *p*<0.05). This is consistent with the higher incidence of splenomegaly in patients with liver disease (89%) when compared to those without (18%) (OR 29.7; 95%CI 9.24 to 84.5; *p*<0.05) (**Table 1**).

### Imaging

66% of patients with liver disease had abnormal liver images, most of which were described as heterogeneous echotexture (**Table 2**). Recently, significantly increased liver stiffness measurement (LSM) transient elastography (FibroScan^®^) has been described in CVID patients with PHTN, showing pathological LSM values >6.5 kPa that should prompt further evaluation (22). In our cohort of patients, transient elastography (FibroScan^®^) higher than 6.5 kPa and abnormal portal doppler ultrasound were observed in 81% and 74% of patients, respectively. However, these tests were not performed in some of the patients with suspected liver disease (50% and 45% respectively).

**Table 2:** Histological changes observed in CVID patients with liver disease and LFTs modification during immunomodulatory treatment.

Hospital	Pt	Histology NRH	Histology Inflammation	LFTs Reduction	Comorbidities requiring immunomodulation	Treatment	Genetic variants
**Centre 1**	1	+		No	CVID enteropathy	Steroids+Tacrolimus+MMF+Ustekinumab	
	2	+					
	3	NA	NA				
	4	+	+	Yes	GLILD	Steroids+MMF+ Rituximab	*NFKB1(*VUS)
	5	+		No	Small lymphocytic lymphoma	Obinutuzumab	
	6	NA	NA				
	7	+	+	Yes	ITP,lymphadenopathy and BM lymphocytic infiltrate	Steroids+MMF	*NFKB1*
	8	+	+	Yes	Lymphadenopathy and BM lymphocytic infiltrate	MMF	*IGGL1 (*VUS)
	9	+	+	No	ITP, neutropenia, lymphadenopathy	High dose of IVIG and steroids	*NFKB1*
	10		+	Yes	Lymphadenopathy	MMF	
	11	+	+	No	AHAI	Steroids	
	12	NA	NA				
	13	NA	NA				
	14	–	+				
	15	NA	NA	Yes	Small bowel lymphoma	R-CHOP	*LRBA (*VUS)
**% Biopsy**	60%	8 (89%)	7 (78%)				
**Centre 2**	1	+	+	Yes	Autoimmune hepatitis	Steroids+Rituximab	
	2		+				
	3		+	No	GLILD	Steroids+ Azathioprine	*CTLA4*
	4		+	Yes	GLILD	Steroids+ Azathioprine+ Rituximab	
	5		+	Yes	CVID enteropathy	Steroids+Infliximab	
	6		+	Yes	CVID enteropathy	Steroids+Infliximab	
	7		+	No	CVID enteropathy	Steroids	
	8		+				
	9		+	Yes	CVID enteropathy	Steroids+Rituximab+Rapamycin	
	10		+	No	CVID enteropathy, AIHA	Steroids+Abatacept	*CTLA4*
**% Biopsy**	100%	1 (10%)	10 (100%)				
**Centre 3**	1	NA	NA	No	ITP	Rituximab	
	2	+					
	3	NA	NA				
	4	+					
	5		+				
	6		+				
**% Biopsy**	67%	2 (50%)	2 (50%)				
**Centre 4**	1	NA	NA				
	2	NA	NA				
	3	NA	NA				
	4	NA	NA	No	GLILD	Rituximab	
	5	NA	NA	Yes	HL	Brentuximab	
	6	NA	NA	No	Large granular lymphocytic T leukemia	Cyclophosphamide	*LRBA, PLCG2* *(*VUS)
	7	+					
**% Biopsy**	14%	1 (100%)	0 (0%)				

CVID, common variable immunodeficiency; NRH, nodular regenerative hyperplasia; LFTs, liver function test; ITP, immune thrombocytopenic purpura; NHL, Non-Hodgkin lymphoma; HL, Hodgkin lymphoma; GLILD, granulomatous-lymphocytic interstitial lung disease; AHAI, autoimmune hemolytic anemia; MMF, mycophenolate mofetil; VUS, variant of unknown significance; IVIG, intravenous immunoglobulin; R-CHOP, Rituximab, Doxorubicin Cyclophosphamide, Vincristine and Prednisone; NA, not available;

### Liver disease complications

Most of the patients had non-cirrhotic PHTN (61%). Complications of PHTN such as ascites, esophageal or gastric varices and gastrointestinal bleeding were observed in 26%, 39% and 8% of patients, respectively. 31% of patients with liver disease showed evidence of liver dysfunction with alteration of blood clotting (**Table 1**).

### Histologic features

**Figure 1:**
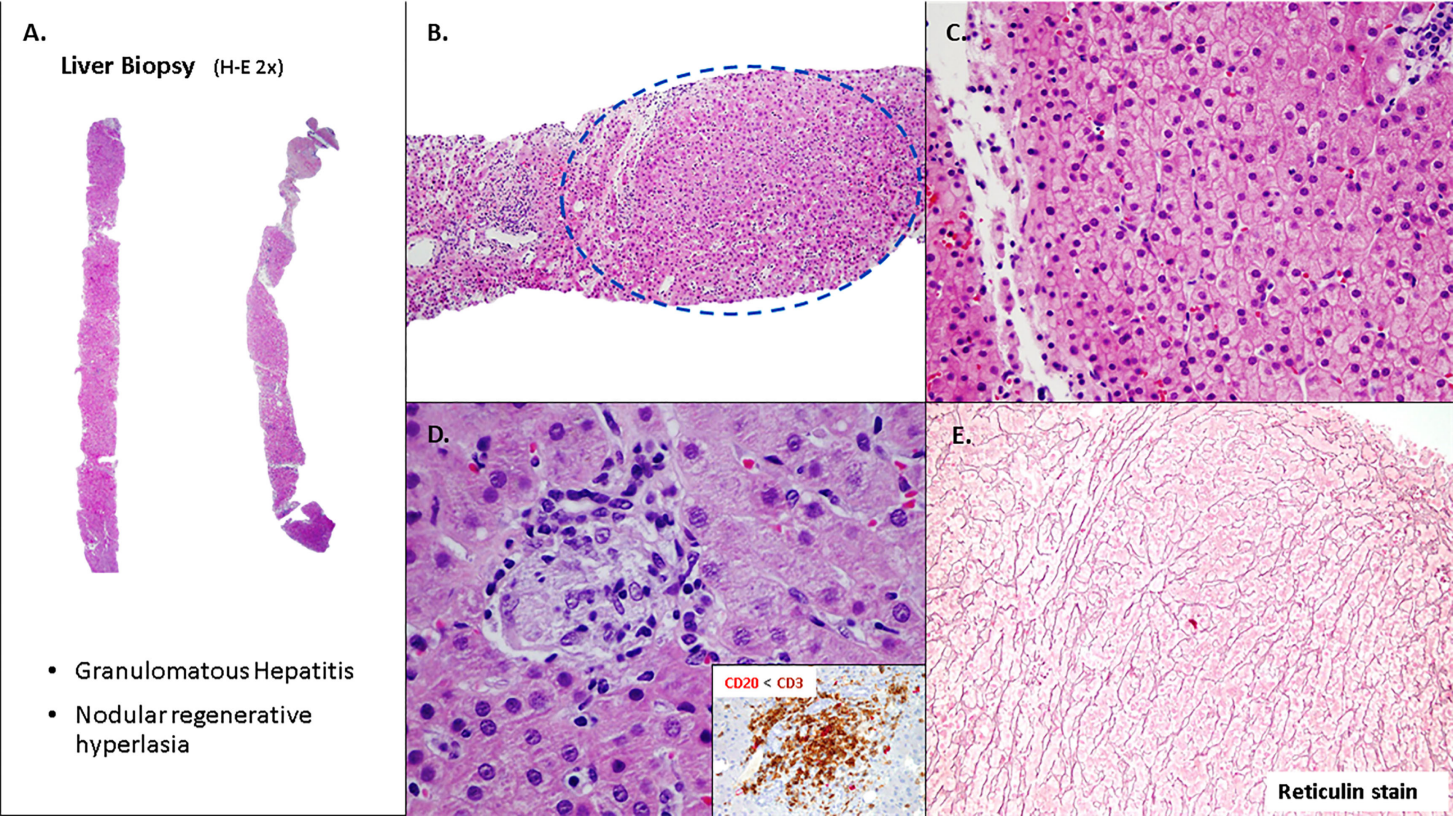
Liver involvement in CVID with nodular regenerative hyperplasia (NRH) and microgranuloma. **(A, B)** Tru-cuts biopsies with nodular appearance. **(C)** Hepatocytes without significant atypia, arranged in trabeculae **(D)**. Focal chronic inflammation with aggregates of histiocytes (microgranulomas), T lymphocytes (CD3+) and scattered B lymphocytes (CD20+). **(E)** Hepatocytes plates are two cells thick and supported by an intact reticulin framework.

**Figure 2:**
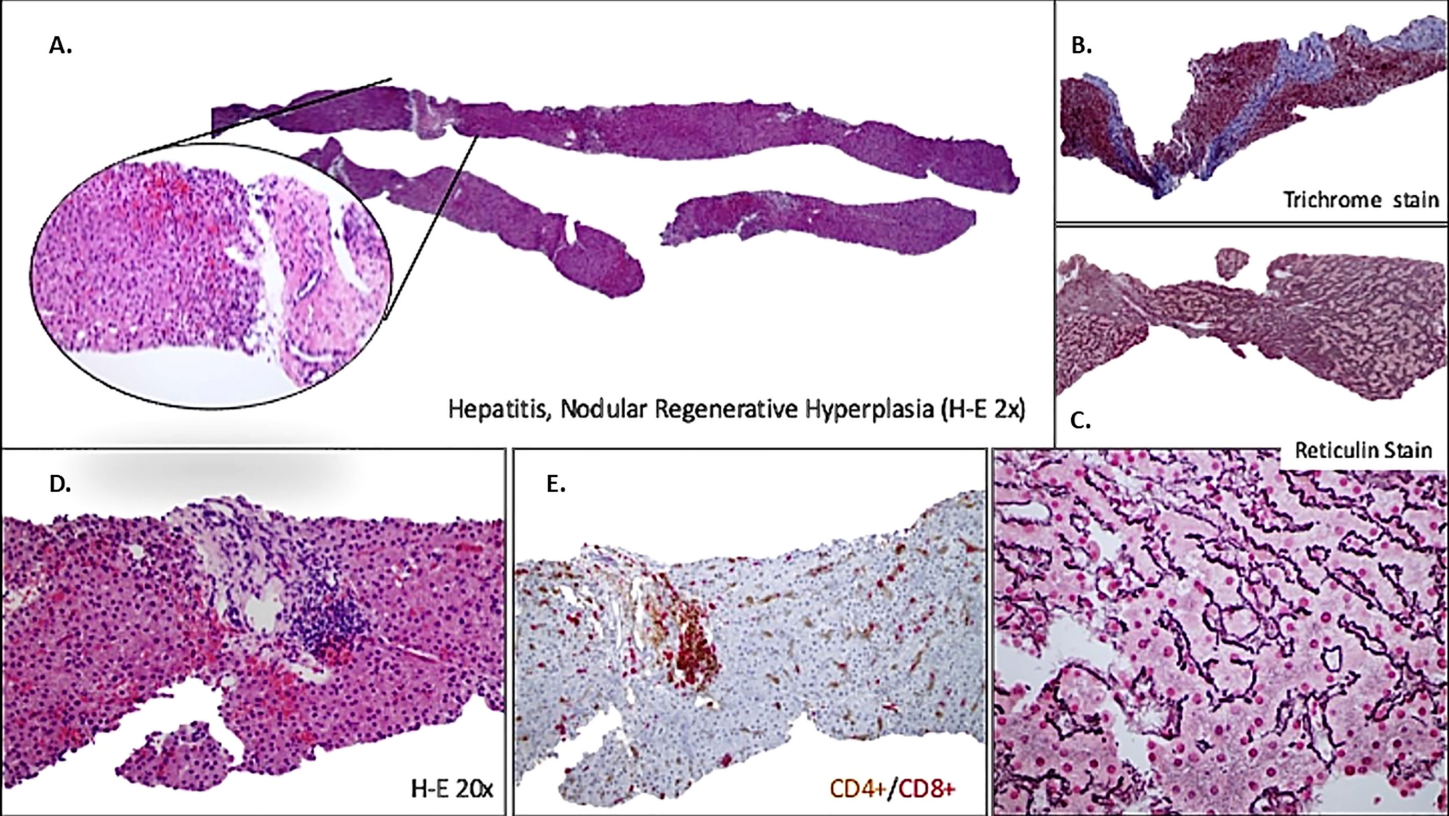
Liver involvement in CVID with nodular regenerative hyperplasia (NRH), inflammatory activity and fibrosis. **(A)** Low-power view showing tru-cut biopsy with vaguely nodular appearance and NRH-like changes. **(B)** Portal-based fibrosis (trichrome stain) affecting entire biopsy. **(C)** Architecture with slight alteration, hepatocytes plates are two cells thick, better seen on the reticulin stain (higher magnification down). **(D)** Portal tracts with lymphocytic infiltrate (minimal interface hepatitis) **(E)** Predominant T lymphocytes with majority expression of CD8+.

Liver biopsy was performed in 66% of patients with liver disease. The most common histological findings included NRH (48%), lymphocytic infiltration (67%), lobular/interface hepatitis (24%), sinusoidal/periportal fibrosis (20%) and granuloma (36%) (**Figures 1**, **2**).

NRH is characterized by the appearance of nodular areas with enlarged hepatocytes, organized into two-cell thick plates alternating with compressed liver cell plates, peri-sinusoidal fibrosis, and focal lymphocytic infiltrates (18). There was a marked difference in the prevalence of NRH between centers (**Table 2**). However, most of the patients had non-cirrhotic PHTN, which is classically associated with NRH.

### Correlation of liver disease and systemic involvement

Overall non-infectious complications such as autoimmune cytopenia, organ specific autoimmune conditions, granulomatous disease, non-infectious CVID enteropathy, lymphoproliferation, among others, occurred in 82% of patients with liver disease compared with 55% of those without (OR 3.45; 95%CI 1.28 to 8.61; *p*<0.05) (**Table 1**).

In this cohort, autoimmune cytopenia was reported in 18% of patients compared with 15% of those without. Non-infectious CVID enteropathy according to biopsy-proven villous atrophy, increased intraepithelial lymphocytosis (IEL), absence of plasma cells and/or follicular lymphoid hyperplasia, increased frequency of CD8+ T cell infiltrates in the intestinal lamina propria, or as inflammatory bowel disease was present in 45% of patients with liver involvement, compared with 29% without (*p=ns*). An infectious aetiology was identified in 39% patients, most commonly campylobacter (29%), giardia (16%) and norovirus (11%).

Lymphoproliferation defined by the presence of persistent lymphadenopathy, polyclonal lymphoproliferative infiltration or lymphoid malignancies occurred in 74% of patients with liver disease compared with 25% without liver disease (OR 4,95; 95%CI 2,02 to 12,26; *p*<0,0005) (**Table 1**). Lymphoid neoplasms were observed in 21% of these patients including B cell, T cell and Hodgkin lymphomas (**Supplemental Table S1**). About half of them had a doubtful histological diagnosis or were classified as lymphoid neoplasm not otherwise specified. Lymphoma is the most frequently reported malignancy in CVID. However, his detection and accurate diagnosis are challenging due to pre-existing polyclonal lymphadenopathy (23) and the presence of oligoclonal T cell populations that has been reported to be increased in patients with CVID (24).

### Immunologic findings

The hallmark of CVID is loss of B cell function. Using the EUROclass system (5) we separated patient groups into those with nearly absent B cells (less than 1%), severely reduced isotype switched memory B cells (less than 2%), and subjects with expansion of CD21(low) B cells (more than 10%).

In keeping with previous reports, phenotyping of B-cell subpopulations has been used to demonstrate a severe reduction of switched memory B cells (CD19+ CD27+IgM-IgD-), defined as less than 2% of CD19+ cells, and expansion of CD21low B cells (more than 10% of B cells) have been associated with autoimmune or inflammatory manifestations (15, 25).

Although the differences were not statistically significant, a higher percentage of patients with liver disease was observed to have expanded CD21low B lymphocytes (63%) and reduced numbers of switched memory B lymphocytes (52%) when compared to patients without liver disease (35%). Again, although 39% of these patients had T lymphopenia, mainly affecting CD4 T cells (less than 400 cell/µl), compared to 31% of patients without liver disease, the differences did not reach statistical significance.

### Correlation of liver disease with CVID course and treatment

55% of the CVID patients with liver disease were treated with immunomodulatory or biologic drugs indicated for the treatment of other autoimmune, inflammatory and lymphoproliferative complications. These drugs included steroids, rituximab, azathioprine, mycophenolate mofetil (MMF), abatacept, tacrolimus, rapamycin, infliximab, ustekinumab, cyclophosphamide and obinutuzumab. There was an overlap of multiple immunomodulators in the treatment received by some patients due to the refractory nature of their disease. Normalization and/or reduction of the LFTs abnormalities during treatment were observed in 52% of these patients, often presenting with progressive worsening of LFTs after treatment discontinuation.

Interestingly, patients treated with rituximab in association with other immunomodulatory drugs (steroids, MMF, azathioprine, rapamycin, among others) showed reduction of LFTs; while patients treated with single drugs such as steroids, rituximab, tacrolimus, ustekinumab, cyclophosphamide and obinutuzumab did not improve the LFTs abnormality during the treatment of different autoimmune/inflammatory complications. Two patients who received infliximab in combination with steroids for the treatment of CVID enteropathy also showed reduction of LFTs. On the other hand, we observed LFTs reduction and/or arrest of their progression in patients receiving monotherapy with MMF and brentuximab for the treatment of lymphoproliferative disease. Two patients with pathogenic variants in the *CTLA4* gene were treated with steroids plus azathioprine and steroids plus abatacept, respectively. None of them showed improvement of the liver profile during treatment (**Table 2**). Three patients showed worsening of the LFTs alteration after treatment with azathioprine, trimethoprim/sulfamethoxazole and chemotherapy for lymphoma (CHOEP regimen). Nevertheless, our cohort of patients with CVID and liver disease is not large enough to achieve a significant correlation between drug combination, comorbidity, histology and LFTs reduction during treatment.

Moreover, no patient in our cohort underwent liver or hematopoietic stem cell transplantation (HSCT). HSCT was ruled out in three patients due to the liver disease itself.

### Mortality

We found differences in mortality between patients with liver disease and those without. The crude mortality rate was 21% and 4%, respectively (OR 7.06; 95%CI 1.50 to 36.08; *p*<0.05). Liver disease was the cause of death in 63% of these patients (**Table 1**).

### Genetic defects

Genetic test including a PID panel by next NGS was performed in 45% of patients with CVID-related liver disease. Probably pathogenic variants in *CTLA4* and *NFKB1* genes were reported in four patients and variants of unknown significance (VUS) in the *NFKB1, LRBA, IGGL1* and *PLCG2* genes were identified in four of them.

### 2-Diagnostic approach, monitoring and management of liver disease in patients with CVID

Eighteen consultants from thirteen Spanish hospitals completed the questionnaire: six immunologists, seven hepatologists, four internal medicine physicians and one paediatrician. The total number of patients currently seen by all participant centres was estimated to be about 578 with a prevalence of liver disease of 24%.

Regarding the follow-up of patients with CVID, there was consensus that liver blood test profile should be requested more than once a year (83% agree) in all patients with CVID. There was no consensus on how often abdominal ultrasound should be performed in the follow-up of these patients (**Table 3**).

**Table 3:** Diagnostic approach, monitoring and management of liver disease in CVID.

	Votes in agreement (%)	Degree of agreement
WORKUP TO SUSPECTED LIVER DISEASE		
Blood test with liver profile	100%	**Unanimity**
Viral load for HBV/HCV	89%	**Consensus**
Abdominal ultrasound	100%	**Unanimity**
Portal doppler ultrasound	94%	**Consensus**
Transient elastography (FibroScan^®^)	83%	**Consensus**
Hepatic hemodynamic (if PHTN is present)	78%	Majority
Liver biopsy	78%	Majority
Endoscopic study (if PHTN is present)	94%	**Consensus**
FOLLOW-UP		
**Follow up of CVID**		
Blood test with liver profile more than once a year	83%	**Consensus**
Abdominal ultrasound every 1-3 years	61%	No consensus
**Follow up of CVID with live disease**		
Blood test with liver profile every 2-4 months	94%	**Consensus**
Abdominal ultrasound every 6-8 months	67%	Majority
Transient elastography (FibroScan^®^) every 12 months	56%	No consensus
Endoscopic study every 12 months	61%	No consensus
MANAGEMENT		
Use of beta-blocker prophylaxis if oesophageal varices are present	83%	**Consensus**
No enough evidence regarding management to prevent progression	89%	**Consensus**
CVID patients with suspected liver disease should be evaluated by a hepatology unit	72%	Majority

CVID, common variable immunodeficiency; PHTN, portal hypertension.

### Diagnostic approach when liver disease is suspected

The majority (77%) of participants agreed that there is no standardized monitoring strategy for the follow-up of liver disease in patients with CVID and that all patients with CVID-related liver disease should be evaluated by a hepatology unit (72% agree).

There was a consensus of 80% or more that the diagnostic workup of CVID-related liver disease should include blood tests with liver profile (100% agree), viral load for HBV and HCV (89% agree), abdominal ultrasound (100% agree), portal doppler ultrasound (94% agree) and transient elastography (FibroScan^®^) (83% agree). The majority agreed that liver biopsy (78% agree) should be essential in the workup of suspected liver disease (**Table 3**).

### Monitoring CVID patients with liver involvement

There was consensus that liver blood test profile should be requested every 2-4 months in patients with CVID that develop liver disease (94% agree). There was no consensus on the frequency of abdominal ultrasound, transient elastography (FibroScan^®^) and endoscopic studies.

There was consensus that an endoscopic study (94% agree) and hepatic hemodynamic (78% agree) should be performed in case of PHTN (**Table 3**).

### Management of liver disease in CVID

There was consensus on the use of beta-blocker prophylaxis for esophageal varices (83% agree) and that there is insufficient evidence on the management of these patients (89% agree). Participants agreed unanimously that there is a need for further development of guidelines for the management of liver disease in CVID (**Table 3**).

## Discussion

In this study we aimed to summarize the evidence on the epidemiology, comorbidities, outcome and treatment of liver involvement in patients with CVID, to develop a consensus statement on the diagnosis and management of these patients.

The majority of patients with CVID and liver disease of our cohort showed an increase of LFTs (76%) and/or radiologic features of liver abnormalities and PHTN (hepatomegaly, splenomegaly, among others) (68%) as initial signs of liver disease. The evolution of liver disease in these patients manifested with chronic abnormal LFTs (95%), thrombocytopenia (79%) in association with hypersplenism, and other PHTN complications over the course of time.

Abnormal liver imaging was present in 66% of those with liver disease, most of which were described as heterogeneous echotexture, consistent with histologic abnormalities of NRH-like changes, inflammation or granulomatous disease. It has been previously described that the presence of NRH and lobular/interface hepatitis, as intrinsic CVID-related liver disease, can be associated with PHTN leading to a poorer prognosis (20). In the absence of clinical reasons for biopsy in most patients, NRH is probably underestimated and may be silent with no clinical consequences for years, but in some cases the pathology leads to PHTN.

Transient elastography (FibroScan^®^) has recently been reported to reveal significantly higher liver stiffness measurement (LSM) in CVID patients with PHTN, showing pathological LSM values >6.5 kPa that should prompt further evaluation (22). FibroScan^®^ was only performed in a proportion of patients with suspected liver disease in our cohort, observing a transient elastography >6.5 kPa and an abnormal portal doppler ultrasound for most of them. Furthermore, there was a notorious difference in the number of biopsies performed and the prevalence of NRH between centres (**Table 2**). This could be explained by the absence of standardized strategies, the presence of poorly representative liver parenchyma in the biopsies (scarce complete portal spaces), and the pathologist variability, as previously described (26). However, most patients had non-cirrhotic PHTN (61%), which is classically associated with NRH. This finding probably reflects the fact that NRH is difficult to diagnose and a high index of suspicion is required to look for it (26).

About 50% of patients with CVID may develop non-infectious inflammatory/autoimmune complications that increase morbidity and worsen survival. Although peripheral B cell phenotypes and the presence of an interferon (IFN) signature have provided some clues about clinical outcomes, the pathogenesis of these complications has not been unravelled (27). It has been shown that if commensal organisms are not appropriately contained, translocation of bacteria and/or their products can drive chronic inflammation. Recent studies reported that circulating bacterial 16S rDNA from intestinal commensal organisms is significantly greater in CVID patients with inflammatory manifestations. It is also associated with increased serum IFN-γ and low numbers of isotype-switched memory B cells (28). We found a low number of switched memory B cells in 52% and CVID-associated enteropathy in 45% of patients with liver involvement. However, the presence of a dysfunctional mucosal barrier could contribute to the translocation of microbial products in CVID, regardless of the presence or absence of known enteropathy in these patients (28).

The pathogenesis of NRH is not fully understood, but CVID patients with NRH are also more likely to present immune-dysregulation related complications (19), suggesting that NRH could represent an immune-mediated manifestation. NRH has been associated with granuloma formation and infiltration of CD8+ cytotoxic T-cells in the liver sinusoids. Studies of infiltrating cells demonstrate predominance of IFN-γ producing T-cells, indicating a possibly T-cell driven origin of NRH. CD8+ T-cell infiltration in the liver may lead to cell-mediated hepatocyte damage, a regenerative process and ultimately vascular abnormalities developing NRH, which has the capacity to progress to PHTN (18, 29, 30).

Non-infectious manifestations occurred in 82% of subjects of our cohort of CVID patients with liver disease. The presence of lymphoproliferation was the predominant non-infectious complication, which was found in 74% of these patients. This could be considered a risk factor for the development of liver disease in CVID. It has been proposed that an altered immune or inflammatory function could be responsible for persistent polyclonal lymphoproliferation (31). The presence of liver disease could be linked to a lymphoproliferative disease and/or there could also be a relation between bacterial translocation and IFN-γ-mediated inflammation in the pathogenesis of NRH and liver involvement in patients with CVID. Future research should improve our understanding of this complex relationship and lead to the development of therapeutic approaches that could attenuate the progression of this liver disorder.

The treatment of immune dysregulation in these patients could be challenging and requires careful balancing of immunosuppression and infectious surveillance. For this reason, the treatment is often delayed. In our cohort, 55% of CVID patients with liver disease received immunomodulatory treatment for different autoimmune/inflammatory and lymphoproliferative disorders, which also reduced the LFTs abnormality in 52% of these patients. However, we do not know whether it could have an impact on the evolution of liver disease. Controlled clinical trials with histological confirmation would be needed to further evaluate this hypothesis. Moreover, liver damage might not be reversible in the end-stage of the disease. An improvement of surveillance and monitoring of CVID complications and the introduction of early targeted therapies for non-infectious phenotypes could prevent the progression of liver disease.

Chronic liver damage may progress to the end-stage of the disease, in which liver transplantation and HSCT are the only therapeutic approaches, even though the serious warnings and poor prognosis of these procedures in CVID patients (3) due to disease recurrence (20) and significant complications and mortality (32, 33). For this reason, HSCT is frequently ruled out in the context of liver disease in patients with CVID. In our cohort, HSCT was dismissed in three patients due to the high risk associated with liver disease complications.

Genetic defects leading to loss of B cell development and other defects in immune regulation have been identified in 20% – 25% of individuals, but the pathogenesis of inflammatory complications in CVID has remained unexplained in most cases (28, 34). Given the heterogeneous nature of CVID, insights regarding molecular mechanisms may uncover specific genetic predispositions to liver disease. However, many patients in this cohort have not been tested. Other potential limitations of the study include the retrospective review, which raises potential concerns about incomplete ascertainment bias. Some patients only went for certain procedures or diagnostic tests if there was an indication that raised clinical suspicion.

We observed that most Spanish centers with experience treating patients with PID do not have a standardized monitoring strategy for the follow-up of liver disease in CVID patients, due to the lack of evidence regarding the management of this complication. This highlights the need to develop international guidelines for the diagnosis and management of patients with CVID-related liver disease.

According to the scientific evidence and the opinion of a broad panel of experts, we recommend that all patients with CVID undergo semi-annual monitoring of LFTs and an annual abdominal imaging study to look for liver anomalies, splenomegaly, or other PHTN features. Patients with any abnormality should be referred to a hepatologist for an evaluation, which should include portal doppler ultrasound, transient elastography, endoscopy and/or liver biopsy. If PHTN is evident, a treatment based on lowering portal pressure and upper gastrointestinal varices should be investigated to reduce morbidity and mortality.

In conclusion, liver disease occurs in a proportion of patients with CVID, mainly in those with non-infectious complications associated with immune dysregulation. Liver disease in CVID is often underreported and underrecognized. It is important for clinicians to be aware of this potential complication to increase screening and to prompt early targeted intervention. Furthermore, this CVID complication may contribute substantially to morbidity and mortality. Multi-site collaborations will be critical to reach sufficient evidence on its management. Further research is needed to evaluate the pathophysiology of liver disease in patients with CVID, as well as to identify personalized treatment options to help prevent the progression of this complication.

## Data availability statement

The original contributions presented in the study are included in the article/**Supplementary Material**. Further inquiries can be directed to the corresponding authors.

## Ethics statement

The studies involving human participants were reviewed and approved by Research Ethics Committee of the Balearic Islands (CEI-IB). Written informed consent for participation was not required for this study in accordance with the national legislation and the institutional requirements.

## Author contributions

VD-C and JP conceived, designed and coordinated the study. VD-C, ML-C, AR, CC, TG, RG-T, CA-M, SS-R, MB-L and MB-V provided clinical data and gave critical advice. VD-C, MS-G and JP analyzed the data and interpreted the results. VD-C and JP wrote the manuscript. MS-G, ML-C and AQ-D gave administrative or technical support. The final version of the manuscript was reviewed by all the co-authors. All authors contributed to the article and approved the submitted version.
