# Supplementary material for: Clinical manifestations and approach to the management of patients with common variable immunodeficiency and liver disease
Source: Front Immunol. 2023 Jun 5;14:1197361. doi: 10.3389/fimmu.2023.1197361 (PMC10277479; doi:10.3389/fimmu.2023.1197361)
Supplement: Supplementary file 1 [file Table_1.docx]

**Supplemental Table S1. Demographic, liver function tests and histologic features in relation to clinical sequelae**

| **Pt** | **Age/Sex** | **ALP**  **(IU/L)** | **AST**  **(IU/L)** | **ALT**  **(IU/L)** | **GGT**  **(IU/L)** | **Total Bilirubin**  **(mg/dL)** | **Progressive/stable LFT alteration** | **Liver histology** | **Sequelae of liver disease** | **Other non-infectious complications** | **Treatment** | **Cause of death** |
| --- | --- | --- | --- | --- | --- | --- | --- | --- | --- | --- | --- | --- |
| 1 | 38  M | 944 | 20 | 27 | 248 | 0.9 | Progressive | NRH | Hepatosplenomegaly, thrombocytopenia | Pancytopenia, CVID enteropathy, lymphadenopathy | Steroids, tacrolimus, MMF, ustekinumab | - |
| 2 | 39  F | 200 | 49 | 56 | 79 | 1.3 | Cyclic | NRH | Thrombocytopenia | CVID enteropathy, lymphadenopathy |  | - |
| 3 | 75  F | 223 | 37 | 33 | 60 | 1 | Progressive |  | Splenomegaly,  esophageal varices, ascites | GLILD, lymphadenopathy, lymphopenia |  | HRS and probable infection |
| 4 | 51  F | 902 | 109 | 85 | 292 | 0.5 | Cyclic | NRH, interface hepatitis, lymphocytic infiltration, granulomas | Hepatosplenomegaly, thrombocytopenia | ITP, lymphopenia, lymphoproliferative syndrome (possible low-grade lymphoma), GLILD | Splenectomy, R-CHOP, Steroids, MMF, rituximab | - |
| 5 | 43  F | 35 | 18 | 13 | 10 | 0.5 | Cyclic | NRH | Hepatosplenomegaly, thrombocytopenia | ITP, neutropenia, lymphopenia, small lymphocytic lymphoma (NHL) | Obinutuzumab | - |
| 6 | 59  F | 72 | 40 | 31 | 102 | 0.5 | Progressive |  | Splenomegaly, thrombocytopenia |  |  | - |
| 7 | 45  M | 198 | 33 | 34 | 71 | 1 | Progressive | NRH, lymphocytic infiltration, periportal fibrosis | Hepatosplenomegaly, esophageal varices, ascites, thrombocytopenia | ITP, neutropenia, diffuse myelofibrosis associated to nodular mature T-cell infiltrates,  (Possible T-cell peripheral lymphoma) | Steroids, GM-CSF/ MMF /QT (CHOEP) | PML |
| 8 | 47  M | 141 | 72 | 119 | 181 | 0.3 | Stable | NRH, lymphocytic infiltration, periportal fibrosis | Hepatosplenomegaly, esophageal varices, thrombocytopenia | Lymphadenopathy  pancytopenia, hypoplastic myelodysplastic syndrome, T lymphocytic infiltrate in the BM | MMF | - |
| 9 | 38  F | 227 | 50 | 57 | 231 | 0.5 | Progressive | NRH, lymphocytic infiltration, periportal fibrosis | Splenomegaly, thrombocytopenia | ITP, neutropenia,  lymphadenopathy | GM-CSF, romiplostin, high dose of IVIG, steroids | - |
| 10 | 75  F | 229 | 29 | 15 | 241 | 1 | Progressive | lymphocytic infiltration | Hepatosplenomegaly, esophageal varices, ascites, thrombocytopenia | CVID enteropathy, lymphadenopathy | MMF | - |
| 11 | 72  M | 194 | 59 | 39 | 283 | 1.5 | Cyclic | NRH, interface hepatitis, lymphocytic infiltration, granulomas | Splenomegaly, esophageal varices, ascites, thrombocytopenia | AIHA, unspecified low grade small B cell NHL | Steroids,  Splenectomy | - |
| 12 | 35  M | 166 | 42 | 47 | 33 |  | Progressive |  | Splenomegaly, thrombocytopenia | CVID enteropathy, lymphadenopathy |  | - |
| 13 | 53  F | 572 | 56 | 63 | 160 | 0.3 | Cyclic |  | Splenomegaly, thrombocytopenia |  |  | - |
| 14 | 57  F | 213 | 16 | 12 | 106 | <1.2 | Progressive |  | - | Lymphadenopathy |  | - |
| 15 | 43  M | 266 | 76 | 91 | 199 | 0.2 | Progressive |  | Splenomegaly | CVID enteropathy, small bowel lymphoma (diffuse large B-cell lymphoma germinal center B-cell-like) | R-CHOP | - |
| 16 | 47  F | 102 | 106 | 167 | 190 | 0.7 | Cyclic | NRH, interface hepatitis, lymphocytic infiltration | Splenomegaly, thrombocytopenia, ascites, autoimmune hepatitis, alteration of blood clotting | - | Steroids, rituximab | - |
| 17 | 71  M | 250 | 176 | 214 | 199 | 1.6 | Progressive | lymphocytic infiltration | Splenomegaly, esophageal varices, ascites, thrombocytopenia, upper gastrointestinal bleeding, alteration of blood clotting | Pancytopenia, AIHA, CVID enteropathy |  | Hepatic failure, CMV disease |
| 18 | 32  M | 201 | 208 | 199 | 109 | 1.9 | Progressive | lymphocytic infiltration, granulomas | Splenomegaly, esophageal varices, ascites, thrombocytopenia, upper gastrointestinal bleeding, alteration of blood clotting | CVID enteropathy, GLILD, lymphadenopathy, Autoimmune thyroiditis, autoimmune atrophic gastritis | Steroids, Azathioprine | Upper gastrointestinal bleeding after TIPS |
| 19 | 58  M | 299 | 108 | 174 | 201 | 0.9 | Cyclic | lymphocytic infiltration | Splenomegaly, thrombocytopenia | CVID enteropathy, GLILD, lymphadenopathy, autoimmune thyroiditis, Autoimmune atrophic gastritis | Budesonide, Steroids, Azathioprine, rituximab | - |
| 20 | 37  M | 180 | 76 | 119 | 190 | 0.8 | Stable | lymphocytic infiltration, granulomas | Splenomegaly | CVID enteropathy, autoimmune thyroiditis, vitiligo, autoimmune atrophic gastritis | Steroids, infliximab | - |
| 21 | 43  F | 278 | 167 | 199 | 2340 | 1.2 | Cyclic | lymphocytic infiltration, granulomas | Splenomegaly, thrombocytopenia | GLILD, lymphadenopathy, autoimmune thyroiditis, vitiligo | Steroids, infliximab | - |
| 22 | 78  M | 185 | 134 | 186 | 210 | 1.5 | Stable | lymphocytic infiltration, granulomas | Splenomegaly, thrombocytopenia, ascites, alteration of blood clotting | CVID enteropathy | Budesonide, Steroids | - |
| 23 | 71  M | 202 | 128 | 147 | 196 | 0,9 | Stable | lymphocytic infiltration | Splenomegaly, thrombocytopenia |  | Budesonide | - |
| 24 | 44  F | 102 | 117 | 190 | 202 | 0.9 | Stable | interface hepatitis, lymphocytic infiltration, granulomas, periportal fibrosis | Splenomegaly, thrombocytopenia, esophageal varices, ascites, alteration of blood clotting | CVID enteropathy | Steroids, rituximab, rapamycin | - |
| 25 | 34  M | 200 | 159 | 145 | 118 | 1,1 | Progressive | lymphocytic infiltration, granulomas, periportal fibrosis | Splenomegaly, thrombocytopenia | CVID enteropathy, NHL,  AIHA, autoimmune atrophic gastritis | Steroids, abatacept | - |
| 26 | 45  F | 226 | 36 | 46 | 153 | <1.0 | Progressive | - | Splenomegaly, thrombocytopenia, esophageal varices | ITP, CVID enteropathy | Rituximab | - |
| 27 | 55  M | 375 | 111 | 62 | 154 | 4,2 | Progressive | NRH | Splenomegaly, thrombocytopenia, esophageal varices, alteration of blood clotting | Pancytopenia |  | HRS |
| 28 | 23  M | 138 | 63 | 57 | 152 | <1.0 | Progressive | - | Splenomegaly, thrombocytopenia, esophageal varices | CVID enteropathy |  | - |
| 29 | 55  F | 154 | 40 | 42 | 108 | <1.0 | Progressive | NRH, granulomas | Splenomegaly, thrombocytopenia, esophageal varices | CVID enteropathy, lymphadenopathy |  | - |
| 30 | 64 | 147 | 20 | 15 | 32 | <1.0 | Cyclic | interface hepatitis | Splenomegaly, thrombocytopenia, esophageal varices, ascites, alteration of blood clotting |  |  | HRS, spontaneous bacterial peritonitis |
| 31 | 48  M | 104 | 30 | 32 | 93 | <1.0 | Progressive | interface hepatitis | Splenomegaly, thrombocytopenia, esophageal varices, upper gastrointestinal bleeding, HCV | CVID enteropathy, Burkitt lymphoma | Burkimab | Lymphoma relapse |
| 32 | 42  F | 69 | 32 | 24 | 42 | 0.5 | Progressive |  | Splenomegaly | lymphadenopathy | Carboplatin | - |
| 33 | 43  M | 122 | 30 | 30 | 52 | 0.5 | Cyclic |  | Splenomegaly, alteration of blood clotting | Pancytopenia, lymphadenopathy |  | - |
| 34 | 60  F | 101 | 50 | 51 | 225 | 0.6 | Progressive |  | Diffuse hepatic steatosis, alteration of blood clotting | CVID enteropathy |  | - |
| 35 | 58  F | 88 | 40 | 26 | 43 | 0.6 | Stable |  | Splenomegaly, thrombocytopenia, alteration of blood clotting, HBV | ITP, CVID enteropathy, GLILD, lymphadenopathy | Rituximab | - |
| 36 | 29  M | 142 | 74 | 79 | 262 | 0,6 | Stable |  | Alteration of blood clotting | Hodgkin lymphoma | OEPA and COPP therapy. Radiotherapy, Brentuximab Vedotin | - |
| 37 | 56  M | 106 | 50 | 42 | 34 | 0.7 | Cyclic |  | Splenomegaly | Large granular lymphocytic T leukemia | Cyclophosphamide | - |
| 38 | 50  F | NA | 55 | 33 | NA | NA | Stable | NRH | Splenomegaly, thrombocytopenia, esophageal varices, alteration of blood clotting | Lymphadenopathy  Cancer |  | Cancer |

Pt: patient; M: male; F: female; LFT: liver function test; ALP: alkaline phosphatise; AST: aspartate trasaminase; ALT: alanine transaminase; GGT: gamma-glutamyl-transpeptidase; NRH: nodular regenerative hyperplasia; GLILD: granulomatous and lymphocytic interstitial lung disease; CVID: common variable immunodeficiency; ITP: immune thrombocytopenic purpura; PML: progressive multifocal leukoencephalopathy; BM: bone marrow; AIHA: autoimmune hemolytic anemia; NHL: Non Hodgkin lymphoma; CMV: cytomegalovirus; HVC: hepatitis C virus; HVB: hepatitis B virus; HRS: hepatorenal syndrome; MMF: mycophenolate mofetil; CHOEP: cyclophosphamide, doxorubicin, vincristine, etoposide and prednisone; GM-CSF: granulocyte-macrophage colony-stimulating factor; TIPS: transjugular intrahepatic portosystemic shunt; OEPA: vincristine, etoposide, prednisolone and doxorubicin; COPP: cyclophosphamide, vincristine, procarbazine and prednisone; R-CHOP: rituximab, doxorubicin cyclophosphamide, vincristine, and prednisone; IVIG: intravenous immunoglobulin; NA: not available.
